# Atrial fibrillation cardiac radioablation target visibility on magnetic resonance imaging

**DOI:** 10.1007/s13246-022-01141-3

**Published:** 2022-06-10

**Authors:** Suzanne Lydiard, Beau Pontré, Boris S. Lowe, Paul Keall

**Affiliations:** 1 ACRF Image X Institute, University of Sydney, 1 Central Avenue, Eveleigh, NSW Australia; 2 Kathleen Kilgour Centre, 18 Twentieth Avenue, Tauranga South, Tauranga, New Zealand; 3 Department of Anatomy and Medical Imaging, University of Auckland, 85 Park Road, Grafton, Auckland, New Zealand; 4 Green Lane Cardiovascular Service, Auckland City Hospital, 2 Park Road, Grafton, Auckland, New Zealand

**Keywords:** Atrial fibrillation, Cardiac, Cardiac radioablation, MRI-guided, Motion management, MRI

## Abstract

Magnetic resonance imaging (MRI) guided cardiac radioablation (CR) for atrial fibrillation (AF) is a promising treatment concept. However, the visibility of AF CR targets on MRI acquisitions requires further exploration and MRI sequence and parameter optimization has not yet been performed for this application. This pilot study explores the feasibility of MRI-guided tracking of AF CR targets by evaluating AF CR target visualization on human participants using a selection of 3D and 2D MRI sequences.MRI datasets were acquired in healthy and AF participants using a range of MRI sequences and parameters. MRI acquisition categories included 3D free-breathing acquisitions (*3D*_*acq*_), 2D breath-hold ECG-gated acquisitions (*2D*_*ECG-gated*_), stacks of 2D breath-hold ECG-gated acquisitions which were retrospectively interpolated to 3D datasets (*3D*_*interp*_), and 2D breath-hold ungated acquisitions (*2D*_*real-time*_). The ease of target delineation and the presence of artifacts were qualitatively analyzed. Image quality was quantitatively analyzed using signal-to-noise ratio (SNR), contrast-to-noise ratio (CNR) and non-uniformity. Confident 3D target delineation was achievable on all *3D*_*interp*_ datasets but was not possible on any of the *3D*_*acq*_ datasets. Fewer artifacts and significantly better SNR, CNR and non-uniformity metrics were observed with *3D*_*interp*_ compared to *3D*_*acq*_.* 2D*_*real-time*_ datasets had slightly lower SNR and CNR than *2D*_*ECG-gated*_ and *3D*_*interp n*_ datasets. AF CR target visualization on MRI was qualitatively and quantitatively evaluated. The study findings indicate that AF CR target visualization is achievable despite the imaging challenges associated with these targets, warranting further investigation into MRI-guided AF CR treatments.

## Introduction

Cardiac radioablation (CR) has prodigious potential to advance the clinical care of those suffering from certain cardiac arrhythmias. Initial clinical CR treatments for ventricular tachycardia, an arrhythmia affecting the lower chambers of the heart, have had very favorable clinical outcomes [1–3]; arrhythmic episodes were reduced, often significantly, in 94% of patients in the first published clinical study [2]. In contrast, initial clinical CR treatments for atrial fibrillation (AF), an arrhythmia predominately affecting the left atrium (LA), have not been as successful; two of the three published clinical treatments resulted in AF recurrence [4, 5]. The differing clinical success may be due to AF presenting additional technical challenges and requirements to CR [6–11], such as having multiple treatment targets that move differentially and lack inherent contrast, complex and often not insignificant target motion, very close target proximity to critical structures, and the need for excellent and high dose target coverage to achieve therapeutic gain. It is hypothesized that magnetic resonance imaging guided (MRIg) AF CR on MRI-linear accelerator hybrid systems (MRI-Linacs) may enable a more efficacious treatment by allowing real-time soft-tissue target visualization, target tracking, and beam-steering during treatment delivery.

The proof-of-concept of MRIg AF CR has been indicated by an experimental phantom study that illustrated the feasibility of using multi-leaf collimator tracking to steer the radiation beam real-time to AF CR targets moving with cardio-respiratory motion [12] and feasibility studies that retrospectively tracked the LA on MRI images of healthy participants and digital phantoms [13, 14]. However, an AF CR target motion characterization study illustrated that the LA does not always move the same as the AF CR targets and therefore may not be a suitable surrogate [6] and a previous planning study illustrated that radiotherapy target margin expansions ≤ 3 mm are likely required to ensure surrounding critical structures are sufficiently protected [15]. To reduce surrogacy errors, there is a desire to investigate the feasibility of MRIg tracking of AF CR targets directly.

MRIg radiotherapy typically utilizes 3-dimensional (3D) MRI of static anatomy with sufficient image quality and spatial resolution to allow accurate target and organ-at-risk delineation and 3D patient alignment [16]. 3D MRI is also often acquired for input into template matching MRIg tracking algorithms. Two-dimensional (2D) and/or 3D cine images showing motion over time may additionally be acquired to evaluate target motion. During treatment delivery on MRI-Linacs, MRIg tracking is usually performed using fast 2D MRI acquisitions for motion monitoring [16], and these acquisitions often sacrifice spatial resolution and/or image quality for high temporal resolution. MRI sequence and parameter optimization for MRIg tracking has been performed for oncology tumors [16], however AF CR targets bring different imaging challenges and requirements.

AF CR targets encompass aspects of the LA and pulmonary veins (PV) at the LA–PV junction [6] and are challenged by geometric complexity, contraction-induced cardiac motion of high and often irregular frequency, respiratory-induced motion, and sometimes turbulent blood flow [6, 17]. For MRI, these challenges often cause low image contrast due to the thin-walled structures, motion artifacts due to cardio-respiratory motion, dark flow artifacts due to sequence susceptibility to B_0_ magnetic field inhomogeneities from turbulent blood flow, off-resonance signal voids due to target proximity to the lungs, spatio-temporal resolution compromises, and often patient motion [17–22]. These complexities also make it difficult to develop phantoms that sufficiently replicate this complex geometry, contrast, deformation, and motion.

Whilst the LA and PVs are often imaged with MRI for diagnostic purposes, MRI sequence and parameter optimization has not yet been performed specifically for MRIg AF CR. This pilot study explores the feasibility of AF CR MRIg treatments by evaluating AF CR target visualization and delineation on human participants using a selection of 3D and 2D MRI sequences. This study specifically explores target visualization in two contexts: (i) the ease of 3D target delineation on 3D MRI datasets is evaluated for the potential use within treatment planning, treatment patient alignment, and/or within real-time tracking algorithms within clinical MRIg AF CR workflows (target visualization_3D_) and (ii) the ease of 2D target visualization on 2D MRI datasets acquired in real-time is evaluated for the potential use in real-time target tracking (target visualization_2Dreal-time_). The findings of this study will support AF CR MRIg development and clinical implementation.

## Methods

**Fig. 1:**
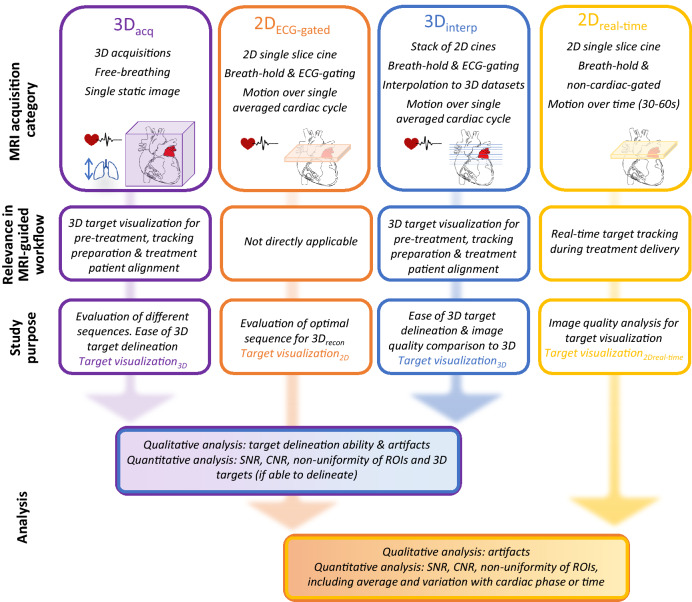
Illustration of overall study method. *CNR* contrast-to-noise ratio, *ROIs* region of interest, *SNR* signal-to-noise ratio

The overall study method is illustrated in Fig. 1.

### Study participants

15 healthy participants (8 male, average age = 39 [range 24–64] years), defined as having no underlying cardiac conditions, and 10 participants diagnosed with AF (8 male, average age = 63 [range 36–73] years) provided written informed consent to undergo non-contrast MRI acquisitions. AF participants exhibited symptomatic, drug refractory, paroxysmal or persistent AF within the 3 months prior to their participation.

### MRI acquisition

Twenty-four participants had a 45–60 min scanning appointment on a 3 T Magnetom Skyra MRI (Siemens, Germany). One healthy participant was scanned on a 1.5 T Magnetom Avanto MRI (Siemens, Germany) to explore the effect of different magnet strengths. All MRI acquisitions were performed using an 18-channel body flex coil with electrocardiogram (ECG) monitoring. No contrast agents were used. Sequence parameters were initially adjusted for individual participants by the magnetic resonance technician as per their standard clinical guidelines for diagnostic cardiac MRI and under the further guidance of a study physicist and cardiologist. Due to MRI scanner time constraints, the selection of MRI sequences and parameters were pragmatically determined for each participant based on the accumulated knowledge from the datasets acquired from prior participants. As seen in Fig. 1, four acquisition categories were pursued; 3D acquisitions (3D_acq_), single-slice electrocardiogram (ECG)-gated 2D acquisitions (2D_ECG-gated_), multiple 2D ECG-gated acquisitions were obtained in multiple breath-holds to allow 3D dataset reconstructions (3D_interp_), and ungated single-slice 2D acquisitions (2D_real-time_). This exploration order was predominantly chosen to follow the MRIg workflow but was also influenced by the study findings as data were acquired and analyzed. Sample sizes for each acquisition category were also guided by study findings as the study progressed. Acquisition categories are described in detail below.

*3D*_*acq*_ Free-breathing 3D MRI acquisitions encompassing at least the LA and PVs were acquired in six healthy participants. Field-of-views ranged from 320–380 mm × 210–250 mm × 50–90 mm for transverse plane acquisitions and 225–250 mm × 300–380 mm × 112–115 mm for sagittal plane acquisitions. Either a radial balanced steady-state free precision (TrueFISP) respiration-navigated sequence was used with a navigator pulse applied at the diaphragm dome and/or a volumetric interpolated examination sequence with radial sampling (StarVIBE), outlined in Table 1. Image acquisition was triggered in end-diastole via the ECG. These two MRI sequences were chosen after an initial exploratory study trialed multiple available cardiac MRI sequences and determined that these were the most promising sequences for AF CR target visualization. As seen in Fig. 1, these datasets were acquired to evaluate target visualization_3D_.

**Table 1:** Summary of free-breathing 3D MRI acquisition sequences (*3D*_*acq*_) and parameters

MRI sequence type	Respiratory navigated TrueFISP					T1 StarVIBE		
Participant	Vol01	Vol02	Vol03	Vol04	Vol06	Vol01	Vol04	Vol05
MRI magnet strength	3 T	3 T	3 T	3 T	1.5 T	3 T	3 T	3 T
In-plane resolution (mm)	0.7 × 0.7	0.7 × 0.7	0.7 × 0.7	0.6 × 0.6	0.6 × 0.6	1.1 × 1.1	0.9 × 0.9	0.9 × 0.9
Slice thickness (mm)	0.6	0.6	0.6	1.0	0.9	1.1	2.8	2.8
Repetition time (ms)	3.2	3.2	3.2	3.4	3.8	2.9	7.6	7.6
Echo time (ms)	1.5	1.4	1.5	1.5	1.6	1.6	2.5	2.5
Flip angle (degrees)	20	25	20	20	90	9.0	9.0	9.0
Acquisition plane	Transverse	Sagittal	Transverse	Transverse	Sagittal	Transverse	Transverse	Transverse
Acquisition time (min)	15.3	20.0	14.1	7.2^a^	10.6	25.2	26.7	26.7

^a^Shorter acquisition time was due to a smaller field-of-view encompassing the left-atria and surrounding pulmonary veins only

*2D*_*ECG-gated*_*:* Transverse, single-slice, breath-held, ECG-gated 2D cine acquisitions were acquired on three healthy participants and one AF participant. This was performed to compare AF CR target visibility on two commonly used 2D cardiovascular MRI sequences, balanced steady-state free precision sequence (TrueFISP) and spoiled-gradient echo fast low angle shot (FLASH). Retrospective ECG-gating was utilized on healthy participants to acquire 25 cardiac phases. ECG-triggering was used in the AF participant due to the potential for variable heart-rhythm. The variation in sequence parameters, as shown in Table 2, were mainly due to patient size variability, patient breath-hold capacity, anatomical variability, and magnet strength. A transverse slice that intersected both target regions was chosen, and care was taken to image a comparable anatomical transverse slice for both True FISP and FLASH acquisitions for each participant. These datasets were acquired to explore optimal 2D sequences for the creation of 3D interpolated MRI datasets. This intermediate data acquisition and analysis step is referred to as target visualization_2D_.

**Table 2:** Summary of 2D MRI acquisition sequences and parameters used for the acquisition of breath-hold, cardiac gated single slice cine images (*2D*_*ECG-gated*_)

MRI sequence type	TrueFISP				FLASH			
Participant	Vol01	Vol03	Vol06	Vol07	Vol01	Vol03	Vol06	Vol07
MRI magnet strength	3 T	3 T	1.5 T	3 T	3 T	3 T	1.5 T	3 T
Participant	Healthy	Healthy	Healthy	AF	Healthy	Healthy	Healthy	AF
ECG-gating	Retrospective	Retrospective	Retrospective	Triggering	Retrospective	Retrospective	Retrospective	Triggering
In-plane resolution (mm)	1.72 × 1.72	1.72 × 1.72	1.25 × 1.25	1.33 × 1.33	1.63 × 1.63	1.63 × 1.63	1.25 × 1.25	1.63 × 1.63
Slice thickness (mm)	6	6	5	6	6	5	5	5
Repetition time (ms)	2.88	2.56	3.03	2.74	5.17	5.35	6.16	3.82
Echo time (ms)	1.41	1.41	1.26	1.51	2.47	2.65	2.74	2.65
Flip angle (degrees)	43	43	59	58	12	10	13	10

All images were acquired in the transverse plane and with retrospective or prospective triggered ECG-gating

*FLASH* spoiled-gradient echo fast low angle shot, *TrueFISP* balanced steady-state free-precision

*3D*_*interp*_*:* For ten healthy and ten AF participants, 8–16, breath-held, contiguous 2D transverse cine acquisitions were acquired to encompass at least the LA and PV ostia using FLASH (3 T, echo time (TE) = 2.65 ms, repetition time (TR) = 3–6 ms, flip angle = 10°, slice thickness = 5 mm, in-plan resolution = 1.4–2 mm). Retrospective ECG-gating was used for healthy participants and ECG-triggering was used for AF participants. 20–25 cardiac phases were acquired, depending on the participant’s breath-hold capacity. To obtain 3D datasets, the multiple cine images acquired for each participant were interpolated into 1 mm voxel static 3D images for each cardiac phase within MIM Maestro (MIM Software Inc., USA). The 3D dataset corresponding to the diastole phase was selected for analysis and is referred to as 3D_recon_. As seen in Fig. 1, these datasets were acquired to evaluate target visualization_3D_.

*2D*_*real-time*_*:* For five healthy and five AF participants, a single breath-held 2D transverse cine was acquired without any cardiac gating using a real-time *TrueFISP* sequence featuring compressed sensing reconstruction (3 T, flip angle = 10°, slice thickness = 5–6 mm, in-plan resolution = 1.5–2.2 mm, 50% sampling, 200–250 ms temporal resolution). Acquisition was continuous until 50–60 image frames were acquired. A transverse slice that intersected both target regions was chosen. This image acquisition is more representative of what could be utilized for MRIg tracking during treatment delivery on an MRI-Linac and these datasets were acquired to evaluate target visuzalization_2Dreal-time_.

### Target definition

Currently no guidelines exist regarding optimal AF CR target definition or delineation. Most pre-clinical and clinical AF CR treatments have utilized target volumes akin to the circumferential lesions created at the pulmonary venous antrum during AF catheter radiofrequency ablation [4–6, 8, 9, 12, 23, 24]. This approach was adopted in this study. Two target volumes were contoured for each participant; the left target volume encircled the left superior- and inferior-PV ostia and the right target volume encircled the right superior- and inferior-PV ostia. For consistency, target delineation was performed by a single observer.

### Image analysis

Target visualization and image quality were qualitatively and quantitatively analyzed to evaluate the feasibility of MRIg AF CR and help determine optimal MRI sequences for AF CR MRIg tracking. Qualitative analysis rated the ease of target visualization and therefore directly explored the study question of whether target visualization and delineation are achievable with MRI. Quantitative image quality analysis provided a less subjective measure that compared the suitability of different MRI acquisition types and allowed image quality evaluation when 3D target delineation was not achievable. Analysis methods were optimized for each MRI acquisition category and study objective.

*Target visualization*_*3D*_* with 3D*_*acq*_* & 3D*_*interp*_*:* The ease of target delineation for each target on each dataset was rated by the single observer performing target delineation on a scale of 1–5 (1 = not able to attempt target delineation due to insufficient image quality or the presence of artifacts in the approximate target region, 2 = able to delineate aspects of the target, but not the entire target, due to poor image quality or the presence of artifacts, 3 = able to approximately delineate the target with low confidence in delineation accuracy, 4 = able to delineate the target but improved image quality would improve delineation confidence and accuracy, 5 = able to delineate the target confidently). The presence of artifacts in or near the target regions were also noted.

**Fig. 2:**
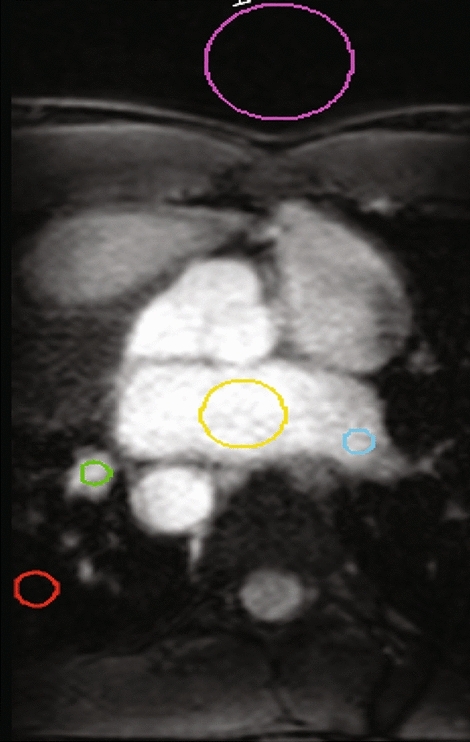
Exemplar illustration of the diameter size and location of ROIs used to quantitatively calculate image quality metrics. Background (*pink*), lung (*red*), left atria *(yellow*), right pulmonary vein region near the target (*green*), left pulmonary vein region near the target (*blue*) ROIs were used

Image quality was quantitatively evaluated using cylindrical region-of-interests (ROIs) of pre-defined volumes and locations. Circular 2D contours of a pre-defined diameter were contoured on each slice until a thickness of 5 mm was achieved. As seen in Fig. 2, the *background* ROI was positioned anteriorly outside the patient, the *lung* ROI was positioned in the posterior aspect of the lung adjacent to the pulmonary veins but excluding cardiac or pulmonary vasculature, the *LA* ROI was positioned centrally within the LA, and the *PV* ROIs were positioned centrally and proximally on the superior or inferior left- and right-PV, whichever was most clearly visible. ROI diameter varied depending on anatomy size and was 35 mm, 10 mm, 20 mm, and 7 mm for *background*, *lung*, *LA*, and *PVs* ROIs respectively. To ensure cylindrical ROI volumes were positioned in comparable 3D anatomical positions for each participant, the most central slice of the LA that also allowed PV visualization was chosen as the central slice of the cylindrical ROIs.

The ease of target visualization was characterized in the context of using template matching for MRIg tracking, a common tracking method in MRIg radiotherapy [25–27]. Template matching is typically easier if at least part of the tracking object is clearly visible, has sufficient contrast compared to directly adjacent tissue, and is of consistent appearance with time. Three metrics were therefore chosen to quantify image quality. The signal-to-noise ratio (SNR) within the *PV* and *LA* ROI’s was calculated, determined as the quotient of the mean ROI intensity and standard deviation of the *background* ROI intensity.

1 $${SNR}_{ROI}= \frac{{Mean\, Intensity}_{ROI}}{{SD}_{background}}$$

The contrast-to-noise ratio (CNR) between the *lung* ROI and *PV* or *LA* ROI was calculated as the quotient between the difference in mean intensity between the evaluated ROI and *lung* ROI and the standard deviation of the *background* ROI intensity.

2 $${CNR}_{lung-ROI}= \frac{{Mean\, Intensity}_{ROI}-{Mean \,Intensity}_{lung}}{{SD}_{background}}$$

Non-uniformity was calculated for the *PV* and *LA* ROIs as the percentage difference between the standard deviation and mean of the intensity within a ROI.

3 $$Non{\text{-}}Uniformity_{{ROI}} = \left( {\frac{{SD_{{ROI}} }}{{Mean\,Intensity_{{ROI}} }}} \right) \times 100\%$$

Analysis was performed for both *PV*s and the *LA* ROIs due to AF CR treatment targets encompassing these regions. CNR was evaluated between *PV* or *LA* ROIs and the *lung* ROI as the blood pool-lung interface is the most distinct contrast boundary surrounding the AF CR treatment targets.

ROI and target delineation was performed within MIM Maestro. Mean and standard deviation intensity values within each evaluated structure were exported from MIM Maestro and quantitative image quality metrics were calculated within Excel (Microsoft, USA). If target delineation was qualitatively rated a score of 4 or 5, image quality metrics were also calculated for the 3D delineated targets using Eqs. 1–3.

*Target visualization*_*2D*_* with 2D*_*ECG-gated*_*:* The presence of artifacts in or near the target regions was noted. Quantitative image quality comparison was performed using 2D ROIs located in the same location as illustrated in Fig. 2. ROI diameters were the same as those for the *3D*_*acq*_ MRI analysis, except for the *LA* ROI, in which a diameter of 15 mm was used to ensure that it was fully encompassed within the LA for all cardiac phases and all participants. The ROIs were positioned in the same anatomical position for both the *TrueFISP* and FLASH datasets for each participant for every image. SNR, CNR, and non-uniformity were calculated as per Eqs. 1–3 for every image. Mean and standard deviation image quality metric values throughout the cardiac cycle were then calculated for each dataset.

*Target visualization*_*2Dreal-time*_* with 2D*_*real-time*_*:* Analysis was as per described above in section *Target visualization*_*2D*_* with 2D*_*ECG-gated*_.

### Statistical analysis

Quantitative image quality results were described as mean ± standard deviation. Significant differences in image quality quantitative metrics between *3D*_*acq*_ versus *3D*_*interp*_, and between *3D*_*interp*_ for healthy participants versus AF participants were calculated using two-tailed Student *t*-tests. Significant differences in image quality quantitative metrics between 2D TrueFISP versus 2D FLASH datasets was calculated using the two-tailed paired Student *t*-test. Statistical analysis was performed in GraphPad Prism version 8 (GraphPad Software, USA). A p-value ≤ 0.05 was considered significant.

## Results

**Fig. 3:**
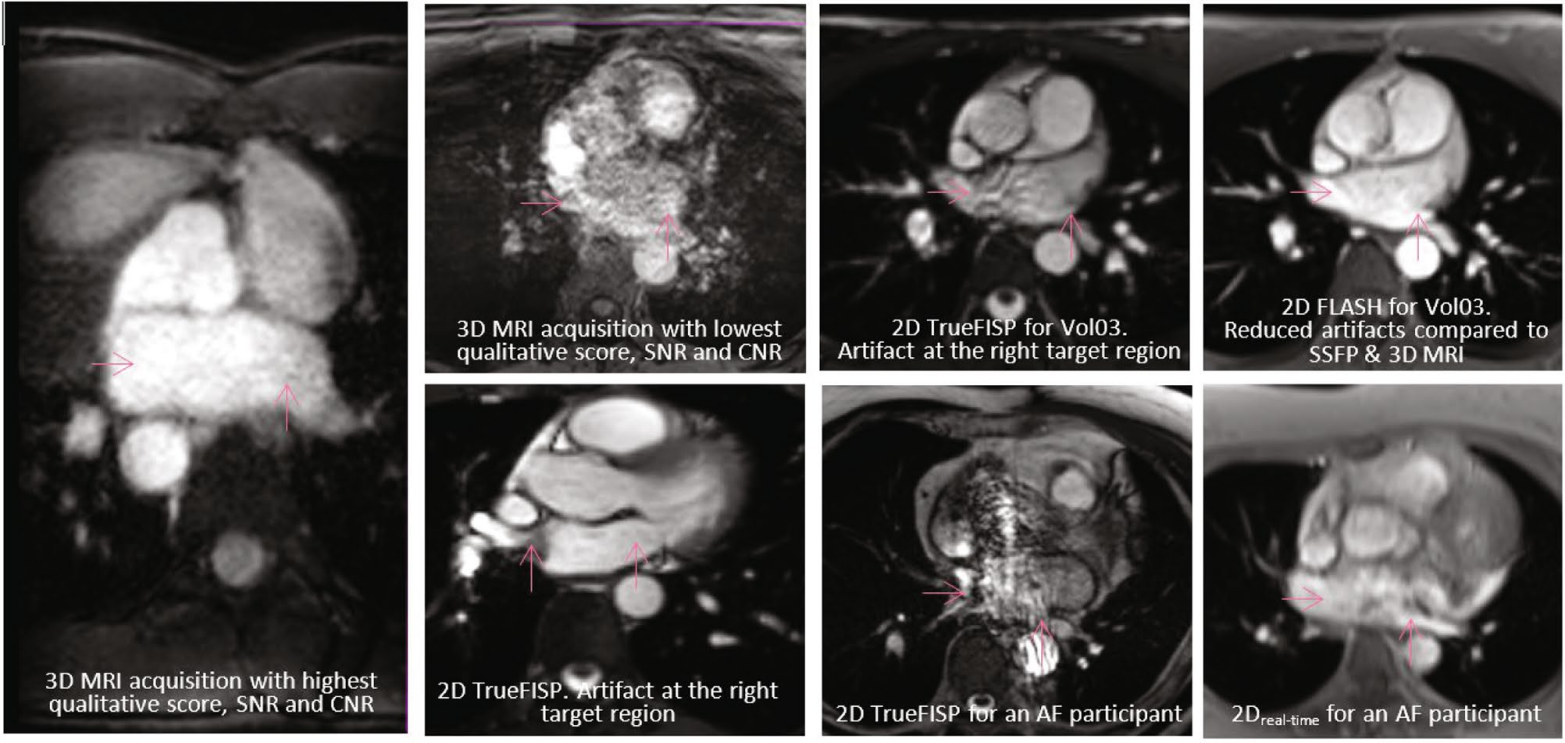
Exemplar images to illustrate the differing image quality and presence of artifacts between MRI acquisition categories and datasets. The best and worst scoring 3D acquisition MRI datasets (*3D*_*acq*_) in terms of qualitative score and quantitative analysis are compared to 2D acquisitions. 2D balanced steady-state free-precision sequence (TrueFISP) and gradient echo fast low angle shot (FLASH) dataset comparisons are shown to illustrate the increased presence of artifacts with TrueFISP. Near real-time 2D acquisition without ECG-gating (*2D*_*real-time*_) datasets had lower signal-to-noise (SNR) and contrast-to-noise (CNR) compared to cardiac-gated datasets (2D TrueFISP & 2D FLASH) and this image quality reduction can be visually observed in these images. Images are for healthy participants rather than atrial fibrillation (AF) participants unless otherwise indicated. SNR and CNR were slightly lower in datasets acquired from AF participants compared to healthy participants. Artifacts detected included motion artifacts, flow artifacts, and signal voids. Arrows indicate the approximate target region

Table 3 displays the cohort-averaged image quality analysis for the analyzed MRI datasets. The average qualitative score for *3D*_*interp*_ was 4.9 with no dataset scoring less than 4, meaning confident target delineation was always possible. In contrast, no *3D*_*acq*_ dataset scored > 3, meaning confident target delineation never occurred. Artifacts were present in all *3D*_*acq*_ datasets, and these included motion and respiratory induced motion artifacts, flow artifacts in the slice plane, and signal voids in the PVs. All *2D*_*ECG-gated*_ datasets acquired with TrueFISP and 80% of *2D*_*real-time*_ datasets had artifacts and these included flow artifacts and signal voids in or near the target regions. In contrast, none of the *2D*_*ECG-gated*_ datasets acquired with FLASH had concerning artifacts in or near the target regions. Exemplar MRI images are shown in Fig. 3 to illustrate comparable image quality and the presence of artifacts.

**Table 3:** Overall image quality analysis results for *3D*_*acq*_ MRI acquisitions (*3D*_*acq*_), 2D single transverse cine acquisitions (*2D*_*ECG-gated*_), 3D datasets derived via interpolation from multiple 2D cine transverse cine acquisitions (*3D*_*interp*_), and near real-time 2D single transverse cine acquisitions without cardiac gating (*2D*_*real-time*_)

Acquisition category	3D_acq_	3D_acq_	2D_ECG-gated_	2D_ECG-gated_	3D_interp_	3D_interp_	2D_real-time_	2D_real-time_
Participants	n = 5H	n = 3H	n = 3H, 1AF	n = 3H, 1AF	n = 10 H	n = 10 AF	n = 5 H	N = 5 AF
Qualitative analysis score [range]	2.0 ± 0.7 [1–3]	2.3 ± 0.6 [2–3]	N/A	N/A	4.9 ± 0.3 [4–5]	4.9 ± 0.3 [4–5]	N/A	N/A
Presence of artifacts	n = 4	n = 2	n = 4	n = 0	n = 1	n = 1	n = 4	n = 4
SNR_PV_left_	46.1 ± 25	70.7 ± 40	211 ± 54	201 ± 69	220 ± 58	196 ± 80	165 ± 40	136 ± 26
SNR_PV_right_	45.5 ± 26	79.2 ± 51	216 ± 27	224 ± 99	216 ± 53	208 ± 83	155 ± 39	135 ± 31
SNR_LA_	95.3 ± 31	74.9 ± 45	243 ± 110	230 ± 100	218 ± 62	205 ± 90	166 ± 46	131 ± 27
SNR_target_average_	N/A^+^	N/A^+^	N/A^#^	N/A^#^	197 ± 51*	185 ± 72*	N/A^#^	N/A^#^
CNR_lung-PV_left_	35.2 ± 28	53.9 ± 40	201 ± 59	194 ± 67	214 ± 55	191 ± 78	161 ± 39	133 ± 26
CNR_lung-PV_right_	34.6 ± 31	62.4 ± 51	206 ± 41	217 ± 98	209 ± 51	202 ± 81	151 ± 37	133 ± 30
CNR_lung-LA_	84.4 ± 33	58.5 ± 50	233 ± 113	223 ± 101	211 ± 59	200 ± 88	162 ± 45	128 ± 27
CNR_target_average_	N/A^+^	N/A^+^	N/A^#^	N/A^#^	190 ± 49*	180 ± 70*	N/A^#^	N/A^#^
Non-uniformity_PV_left_	29.7 ± 14	16.6 ± 4.5	14.3 ± 5.7	13.7 ± 5.0	4.94 ± 1.1	6.23 ± 2.5	6.02 ± 2.7	5.14 ± 2.1
Non-uniformity_PV_right_	26.2 ± 13	18.4 ± 13	19.6 ± 14	7.68 ± 1.7	4.83 ± 1.3	6.36 ± 2.1	6.20 ± 3.7	4.93 ± 3.7
Non-uniformity_LA_	10.8 ± 4.0	10.0 ± 3.0	10.9 ± 6.6	13.7 ± 5.0	7.27 ± 1.7	8.62 ± 2.4	7.51 ± 4.0	7.85 ± 2.9
Non-uniformity_target_average_	N/A	N/A	N/A	N/A	17.5 ± 3.6	18.3 ± 6.6	N/A	N/A

Cohort-average ± standard deviation is given for each image acquisition type for both qualitative and quantitative analysis. The qualitative analysis score defined the ease of target delineation with 1 = not able to attempt target delineation due to insufficient image quality in the approximate target region and 5 = able to delineate the target confidently. Signal-to-noise ratios (SNR), contrast-to-noise ratios (CNR) between the evaluated structure and the surrounding lung tissue, and non-uniformity metrics were quantitatively analysed for pre-defined regions-of-interests. If an image received a qualitative score ≥ 4, SNR, CNR and non-uniformity metrics were also calculated for the 3D left and right targets and average values for both targets are provided.

*H* healthy participant, *AF* participant diagnosed with atrial fibrillation

*100% of datasets received a qualitative score ≥ 4

^+^0% of datasets received a qualitative score ≥ 4

^#^3D target delineation not possible due to datasets being a single 2D cine image slice

**Fig. 4:**
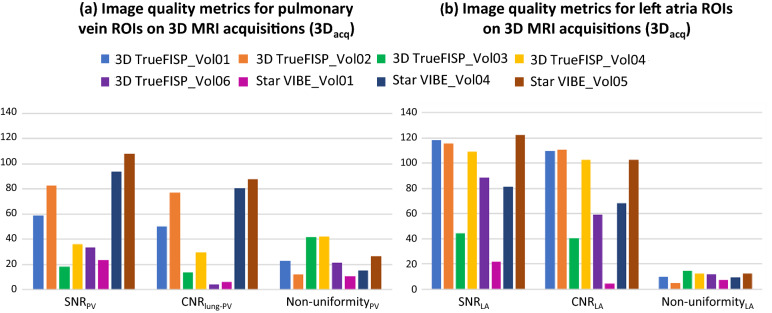
Signal-to-noise ratio (SNR), contrast-to-noise ratio (CNR) between the evaluated ROI(s) and lung ROI, and non-uniformity image quality metrics for *3D*_*acquisition*_ datasets. Results are given for the average of the left and right PV ROIs (**a**) and for the left atria (**b**)

**Fig. 5:**
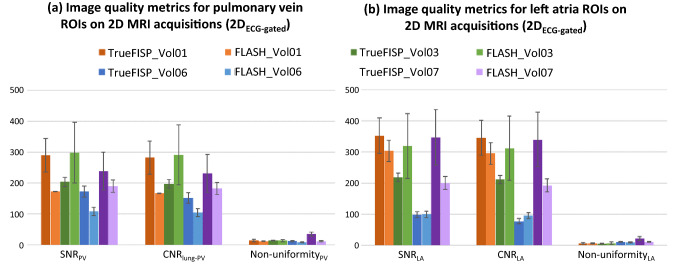
Signal-to-noise ratio (SNR), contrast-to-noise ratio (CNR) between the evaluated ROI(s) and lung ROI, and non-uniformity image quality metrics for *2D*_*ECG-gated*_ datasets. Results are given for the average of the left and right PV ROIs (**a**) and for the left atria (**b**). The bars and error bars represent the average and standard deviation of the image quality metric throughout the cardiac cycle respectively

**Fig. 6:**
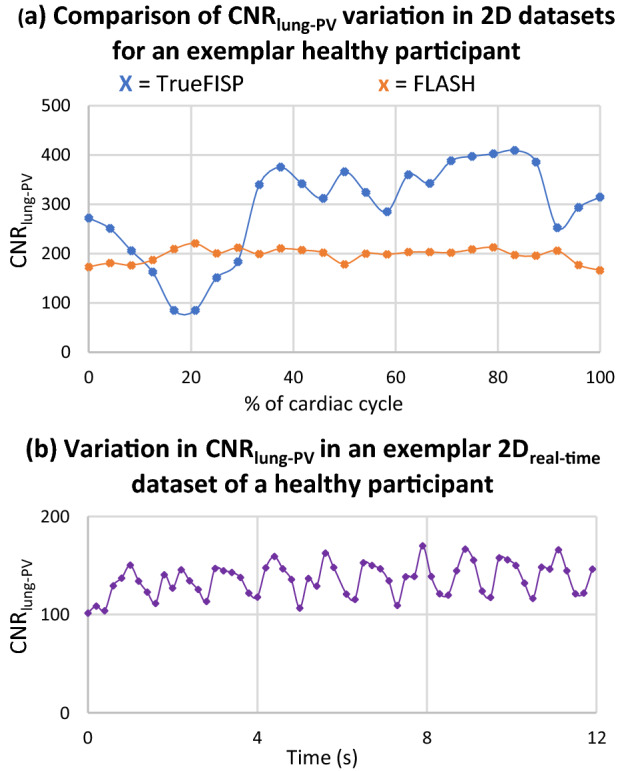
Variation of average contrast-to-noise ratio (CNR) between the two PV ROIs and surrounding lung ROI **a** throughout the cardiac cycle for balanced steady-state free-precision sequence (TrueFISP) in blue and gradient echo fast low angle shot sequence (FLASH) in orange for participant Vol03 and **b** with time for an exemplar 2D_real-time_ dataset. The larger variation in CNR in TrueFISP compared to FLASH observed in **a** and the periodic variation observed in **b** loosely corresponding to heart-rate were typical of what was observed in other participants

As seen in Table 3, SNR and CNR were lower in *3D*_*acq*_ compared to *3D*_*interp*_ datasets_*.*_ The variability in image quality metrics seen in *3D*_*acq*_ datasets, as seen in Fig. 4, is likely due to variations in acquisition sequences and parameters as well as participant variability. Quantitative image quality metrics were comparable between TrueFISP and FLASH *2D*_*ECG-gated*_ datasets (p > 0.05). However, there was greater variation in SNR and CNR throughout the cardiac cycle for 2D TrueFISP compared to 2D FLASH when analyzing and comparing the same anatomical 2D plane, as seen in Figs. 5 and 6. Due to the presence of artifacts and variability in image quality metrics throughout the cardiac cycle in TrueFISP, FLASH was chosen as the best available sequence for acquiring multiple 2D cine images for *3D*_*recon*_. *3D*_*interp*_ gave significantly better values for all evaluated quantitative image quality metrics compared to *3D*_acq_ (p ≤ 0.001). All evaluated *3D*_*interp*_ image quality metrics were comparable (p > 0.05) between healthy and AF participants, although it was qualitatively observed that AF participants generally had noisier images than healthy participants, as seen in Fig. 3. SNR and CNR in *2D*_*real-time*_ appeared to fluctuate periodically, as shown in Fig. 6b, likely caused by cardiac contraction.

## Discussion

This pilot study evaluated AF CR target visualization on a variety of MRI acquisitions to explore the feasibility of MRIg AF CR treatments. MRIg tracking via template matching usually requires (i) 3D target delineation on 3D MRI datasets acquired pre-irradiation for tracking set-up and patient alignment, as well as (ii) target visualization and tracking on 2D MRI images acquired during treatment delivery. This study explored the ease of MRI target visualization for both applications. (i) *Target visualization*_*3D*_*:* 3D datasets derived from multiple 2D FLASH MRI acquisitions (*3D*_*interp*_) exhibited sufficient image quality for confident 3D target delineation and therefore present a plausible pre-irradiation 3D MRI dataset acquisition method. The 3D MRI acquisitions explored in this study (*3D*_*acq*_) did not provide sufficient image quality for confident 3D target delineation. 2D FLASH provided comparable image quality to the more commonly used 2D TrueFISP cardiac MRI sequence and had fewer artifacts and variability in image quality metrics with cardiac contraction. (ii) *Target visualization*_*2Dreal-time*_*: 2D*_*real-time*_ datasets, which were more representative of MRI acquisitions for MRIg tracking during treatment delivery, had lower SNR and CNR compared to cardiac-gated 2D MRI datasets, however image quality appeared reasonable. The findings for target visualization_3D_ and target visualization_2Dreal-time_ combined conclude that AF CR target visualization for MRIg AF CR is achievable on currently available commercial MRI sequences despite the imaging challenges associated with these targets. Further investigation of MRIg AF CR treatments therefore appears warranted provided MRI acquisitions are carefully selected.

TrueFISP sequences are commonly used in diagnostic cardiac MRI because they provide excellent SNR and superiorly enhances the contrast between the blood pool and adjacent myocardium compared to simpler gradient-echo sequences such as FLASH [19, 21]. AF CR treatment targets often encompass the blood pool within the PVs and LA and therefore distinction between the blood pool and myocardium is not essential for this specific application. This study illustrated that FLASH provides superior image quality consistency with time and a reduction in imaging artifacts in the AF CR target region, attributes that are generally more desirable for MRIg. *2D*_*real-time*_ datasets were acquired using an TrueFISP acquisition due to this sequence having the most suitable temporal resolution out of the commercially available sequences available on the utilized MRI scanner. Ungated, real-time FLASH sequences may offer fewer artifacts in the AF CR target region and this warrants further investigation. The variation in SNR and CNR with time on *2D*_*real-time*_ datasets also warrants further investigation; this variation may challenge template matching based MRIg tracking but may also potentially offer a simple method of detecting systole and diastole for incorporation into template matching based MRIg tracking utilizing multiple tracking templates.

Artifacts and sequence limitations detected in this study are consistent with what has been previously described in studies utilizing non-contrast MRI to visualize the PVs for diagnostic or pre-catheter ablation imaging [17, 19]. Whilst previous research describe comparable artifacts, this pilot study was the first to specifically evaluate the impact of these artifacts and image quality on AF CR target delineation in the context of MRIg AF CR. Gadolinium contrast enhanced MRI is usually used for identifying PV and LA anatomy prior to catheter ablation [28]. This study only explored non-contrast MRI sequences because the use of contrast agents is challenging for lengthy MRIg treatment deliveries and the use of contrast in pre-irradiation imaging and not during irradiation could cause tracking difficulties.

MRIg AF catheter ablation is a developing treatment that has been advancing in parallel to MRIg CR. MRIg catheter ablation procedures typically utilize navigator-gated whole heart SSFP sequences to create 3D surface mesh reconstructions of the cardiac cavities and large thoracic vessels and real-time MRI images for catheter localization via signal loss or hyper amplification within MRI images or microcoils integrated within catheter tips [29]. The real-time MRI learnings from MRIg catheter ablation are therefore not directly applicable to non-invasive MRIg AF CR as the use of catheters or metal fiducials are not desired. The target visualization_2Dreal-time_ findings from this study may be of interest to the MRIg catheter ablation community if they desire to locate the soft-tissue ablation target directly. This study explored 3D target visualization for the specific purpose of AF CR treatment planning, treatment patient alignment, and/or for incorporation within real-time target tracking algorithms such as 3D–2D template matching. The findings are therefore unique to MRIg AF CR and had not previously been directly explored in prior MRIg catheter ablation studies. Due to CR planning and delivery processes, only non-contrast MRI sequences acquired in the MRI magnet planes, as opposed to anatomical planes, were explored. This study evaluated the visualization of the CR target itself to aid 3D–2D template matching tracking algorithms rather than the external cardiac chamber or vessel surfaces and this potentially poses a more challenging task for MRIg CR than MRIg CA due to the lack of inherent contrast between the ‘target’ and surrounding tissue.

MRI sequence and parameter optimization has been previously described for MRIg tracking of tumours, but it is acknowledged that ideal sequences, sequence parameters, and slice orientations are likely to be entity and patient-specific [16], warranting the exploration into MRI sequence optimization for AF CR targets. Transverse slice acquisitions were predominantly used in this pilot study to enable image quality analysis for both AF CR target regions on 2D MRI and because preliminary imaging indicated that aliasing artifacts were common with coronal 2D acquisitions. It has been proposed that a sagittal acquisition plane may improve cardiac MRI image quality [17], however, the method of using transverse 2D cine images in this study allowed confident target delineation in all *3D*_*interp*_ datasets.

This study is limited by its small sample size and exploratory nature. However, the importance of pilot studies in the overall research process is recognized despite the inherent limitations of pilot studies [30]. MRI scanner availability and patient tolerability constraints did not allow for systematic sequence and parameter optimization and MRI sequence and parameters were therefore pragmatically determined for individual participants based on the findings from prior participants. The authors believe that utilizing human participants in this study provided more useful findings compared to a phantom study that may have enabled more systematic sequence and parameter optimization but be limited by phantom simplicities and approximations. This exploratory study provides clear testable hypothesis with specific types of acquisitions, which could be explored further in future studies.

Further study limitations include the fact that diagnostic MRI scanners were used rather than MRI-Linacs and a 3 T scanner was mostly used, which is a higher magnet strength than what is currently utilized in commercially available MRI-Linacs. One participant was scanned on a 1.5 T scanner, the same magnet strength that is used in one commercial MRI-Linac system, and the image quality for this participant was comparable to the participants scanned on the 3 T scanner. In some cases, particularly cardiac imaging, lower magnet strengths may be preferable [31, 32], however, conclusions cannot be made regarding this from this study due to the very small sample size. The authors feel that the results of this study are broadly applicable to MRIg AF CR, particularly considering imaging capabilities on commercial MRI-Linacs are variable and rapidly developing. Utilizing commercially available MRI sequences on diagnostic MRI scanners provides insight into optimal sequence types and provides an upper-limit on achievable image quality to guide MRI-Linac technology development.

By illustrating that AF CR target visualization on MRI is achievable, this study answered an important initial question that encourages the future development of MRIg AF CR. The findings from this study have allowed AF CR target motion to be characterized, which has been evaluated separately due to differing study objectives [6] and could aid the development of AF CR MRIg tracking algorithms [33]. To realize and evaluate the full potential of MRIg tracking of AF CR targets, comparable analysis should be performed on one or several MRI-Linac systems, tracking algorithm performance dependency on image quality should be analyzed, and optimized real-time AF CR MRIg target tracking algorithms and workflows need to be demonstrated on MRI-Linacs.

## Conclusion

This pilot study qualitatively and quantitatively evaluated MRI AF CR target visualization. Confident target delineation was achievable on 3D datasets derived from multiple 2D MRI acquisitions but was not achievable on 3D MRI acquisitions. SNR and CNR were more consistent throughout the cardiac cycle on 2D MRI datasets acquired with FLASH compared to TrueFISP. The study findings indicate that AF CR target visualization is achievable despite the imaging challenges associated with these targets, warranting further investigation into MRI-guided AF CR treatments.
